# Conserved Role of *unc-79* in Ethanol Responses in *Lightweight* Mutant Mice

**DOI:** 10.1371/journal.pgen.1001057

**Published:** 2010-08-12

**Authors:** David J. Speca, Daisuke Chihara, Amir M. Ashique, M. Scott Bowers, Jonathan T. Pierce-Shimomura, Jungsoo Lee, Nusrat Rabbee, Terence P. Speed, Rodrigo J. Gularte, James Chitwood, Juan F. Medrano, Mark Liao, James M. Sonner, Edmond I. Eger, Andrew S. Peterson, Steven L. McIntire

**Affiliations:** 1 Department of Neurology and the Ernest Gallo Clinic and Research Center, University of California San Francisco, Emeryville, California, United States of America; 2 Department of Statistics, University of California Berkeley, Berkeley, California, United States of America; 3 Department of Animal Science, University of California Davis, Davis, California, United States of America; 4 Department of Anesthesia and Perioperative Care, University of California San Francisco, San Francisco, California, United States of America; Harvard Medical School, United States of America

## Abstract

The mechanisms by which ethanol and inhaled anesthetics influence the nervous system are poorly understood. Here we describe the positional cloning and characterization of a new mouse mutation isolated in an N-ethyl-N-nitrosourea (ENU) forward mutagenesis screen for animals with enhanced locomotor activity. This allele, *Lightweight* (*Lwt*), disrupts the homolog of the *Caenorhabditis elegans (C. elegans) unc-79* gene. While *Lwt/Lwt* homozygotes are perinatal lethal, *Lightweight* heterozygotes are dramatically hypersensitive to acute ethanol exposure. Experiments in *C. elegans* demonstrate a conserved hypersensitivity to ethanol in *unc-79* mutants and extend this observation to the related *unc-80* mutant and *nca-1;nca-2* double mutants. *Lightweight* heterozygotes also exhibit an altered response to the anesthetic isoflurane, reminiscent of *unc-79* invertebrate mutant phenotypes. Consistent with our initial mapping results, *Lightweight* heterozygotes are mildly hyperactive when exposed to a novel environment and are smaller than wild-type animals. In addition, *Lightweight* heterozygotes exhibit increased food consumption yet have a leaner body composition. Interestingly, *Lightweight* heterozygotes voluntarily consume more ethanol than wild-type littermates. The acute hypersensitivity to and increased voluntary consumption of ethanol observed in *Lightweight* heterozygous mice in combination with the observed hypersensitivity to ethanol in *C. elegans unc-79*, *unc-80*, and *nca-1;nca-2* double mutants suggests a novel conserved pathway that might influence alcohol-related behaviors in humans.

## Author Summary

Alcoholism is costly to both individuals and society. Studies of twins indicate that there is a substantial genetic component to this disease; however, it has been difficult to identify underlying genetic factors in humans, in part because of the small effect of any one gene. Because of their genetic uniformity, subtle effects on behavior of single gene deletions can be detected in inbred mice and used to model human disease. Here we describe the identification of a new mouse mutant, named *Lightweight*, with altered response to alcohol and inhaled anesthetics. *Lightweight* mice are dramatically hypersensitive to a high-dose injection of alcohol. In addition, when given a choice, *Lightweight* mutants voluntarily consume more alcohol than non-mutant animals. *Lightweight* mice may provide novel insight into the mechanism of action of alcohol, and studies of this gene in humans may lead to a better understanding of alcoholism and its treatment.

## Introduction

Alcohol is enjoyed by many, but its abuse can lead to enormous adverse individual and societal consequences. According to the World Health Organization, alcohol abuse accounts for 4% of the global health burden [1]. While twin, adoption, and family studies suggest that there is a strong genetic component to alcoholism [2], [3], identifying susceptibility factors in human populations is difficult because of the heterogeneity of the disorder and the likelihood that there are multiple genes of small effect that contribute to the disease.

Invertebrate genetic screens have identified several genes with clear effects on response to ethanol and inhaled anesthetics. In *Caenorhabditis elegans (C. elegans)*, for example, *unc-79* (and *unc-80*) mutants are hypersensitive to the immobilizing effects of halothane and other anesthetic agents [4], [5]. In addition, *unc-79* mutants are also reported to have altered responses to the immobilizing effects of ethanol [6]. In *Drosophila melanogaster (Drosophila)*, Krishnan and Nash identified an allele of the *narrow abdomen* (*na*) gene in a forward mutagenesis screen for halothane sensitivity [7]. The *na* gene product has the predicted topology of a voltage-gated cationic channel [8] but efforts to characterize it electrophysiologically were unsuccessful until recently when Ren and colleagues proposed that the mouse homolog of this channel, which they named NALCN, was a tetrodotoxin-insensitive, voltage-independent cationic (leak) channel that may be critical for altering the resting membrane potential of neurons [9]. A mouse homozygous knockout allele of the NALCN gene was perinatal lethal, perhaps due to a respiratory defect [9].

Numerous studies suggest that *unc*-79, *unc*-80, and NALCN, function in the same biochemical pathway. In both *C. elegans* and *Drosophila*, for instance, mutants exhibit altered locomotor behavior (“fainting” in worms and hesitant walking in flies) and have altered responses to anesthetics [10]. In addition, the protein expression of these gene products is interdependent, i.e. disruption of *unc-79* or *unc-80* also yields lower or absent expression of NALCN orthologs and vice versa, leading to the hypothesis that they function as a complex [10]–[14]. Most compelling is recent data demonstrating that the function of the NALCN protein can be modulated by the peptide neurotransmitters substance P and neurotensin and that the *unc-80* gene product is required to mediate this signal transduction pathway [13], [14]. Others have demonstrated *in vitro* that the M3 muscarinic receptor can activate the NALCN channel [15]. Together, these data indicate that the NALCN channel and associated proteins may be responsible for a ‘slow’ excitation that can be evoked by substance P, neurotensin, acetylcholine or norepinehprine [16]–[18].

Not only has forward mutagenesis been an invaluable approach in *C. elegans* and *Drosophila* for the study of the nervous system, more recent forward mutagenesis efforts in mice have isolated numerous mutants that influence a variety of behavioral phenotypes [19]–[22]. We previously performed a mouse forward mutagenesis screen using a sensitized genetic background based on a heterozygous null mutation in the dopamine transporter (DAT) to enrich for dominant mutations that enhance dopaminergic neurotransmission [23]. This screen successfully identified five loci that influence locomotor behavior in a quantitative manner, two of which were *dependent* on the sensitized background and three of which acted *independently* of the sensitized background. Here we report that the dominant behavioral phenotype of one of the *independent* loci, *Lightweight (Lwt)*, results from a nonsense mutation in the mouse homolog of the *C. elegans unc-79* gene. *Lightweight* heterozygotes are mildly hyperactive and have altered response to ethanol and inhaled anesthetics, consistent with a conserved function of the mammalian unc-79 gene product.

## Results

### Mapping and positional cloning of the *Lightweight* mutation

**Figure 1:**
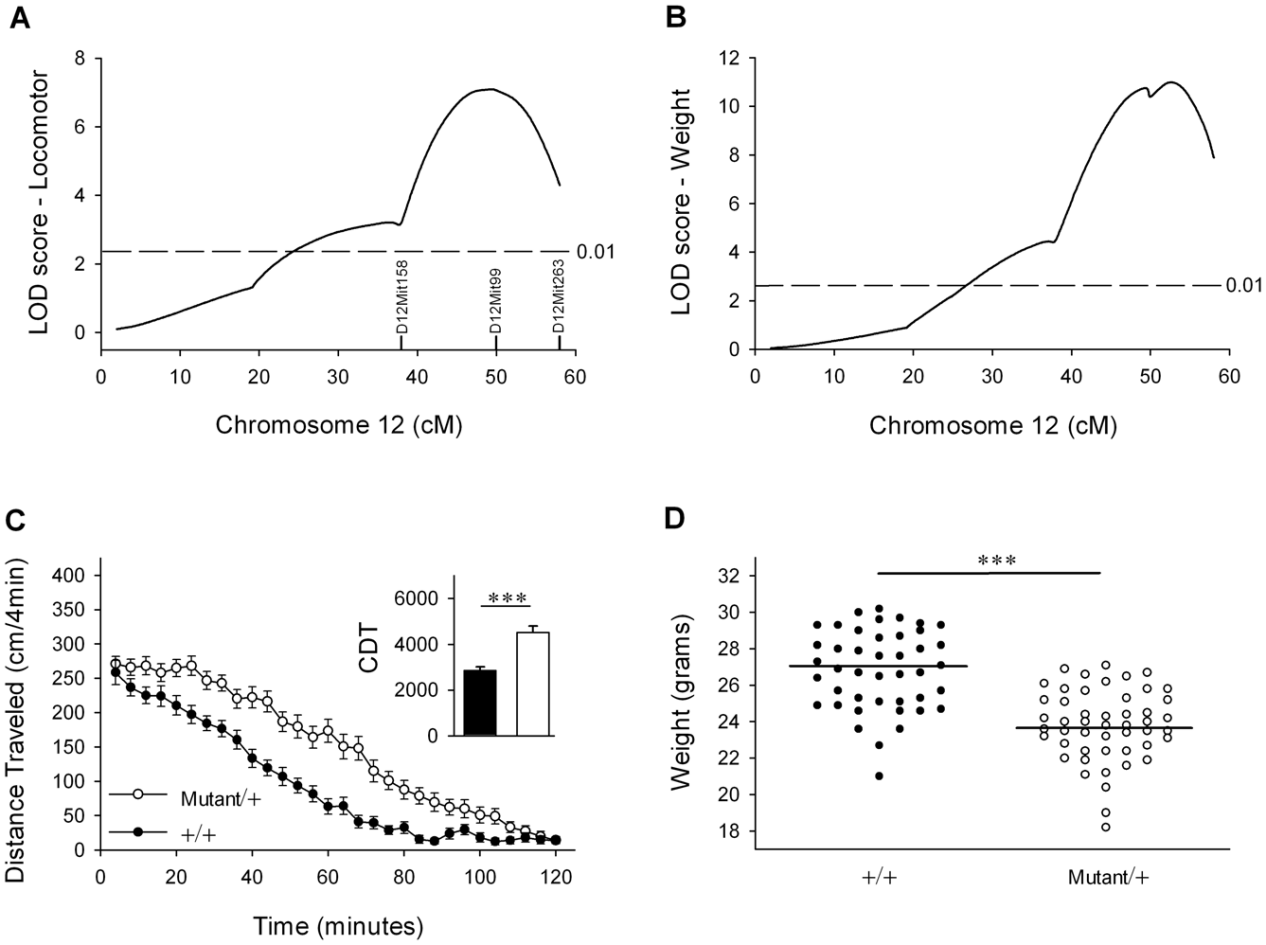
An ENU-induced QTL for locomotor activity and body weight maps to distal mouse chromosome 12. (A) A mutagenized (B6:D2^*^) male 28C4 was backcrossed to C57BL/6J wild-type females and spontaneous locomotor activity of male progeny was monitored for a period of two hours followed by QTL analysis (see reference [23] for details). Significant (*P*<0.01) levels of linkage (dashed black line) were determined by permutation testing [39]. (B) Male B6:D2*×B6 progeny were weighed between the age of 8 to 10 weeks and QTL analysis was performed. (C) Haplotype analysis of the mapping population indicates that Mutant/+ mice, which are unambiguous carriers of mutagenized chromosome 12 across the 95% confidence interval (n = 50), were modestly hyperactive relative to +/+ animals (n = 43) across the same confidence interval. *Inset:* Cumulative distance traveled (CDT) over two hours (in centimeters) is significantly elevated in Mutant/+ males (white bar) versus +/+ animals (black bar) (***P≪0.0001, Student's t-test). (D) Scatterplot of body weight haplotypes of unambiguous +/+ males (•, n = 43) and Mutant/+ males (**○**, n = 49) across the 95% confidence interval on chromosome 12. Black line indicates average weight of population. +/+ males weigh significantly more than Mutant/+ males (***P≪0.0001, Student's t-test). See Figure 2 for haplotype structure of Mutant animals.

**Figure 2:**
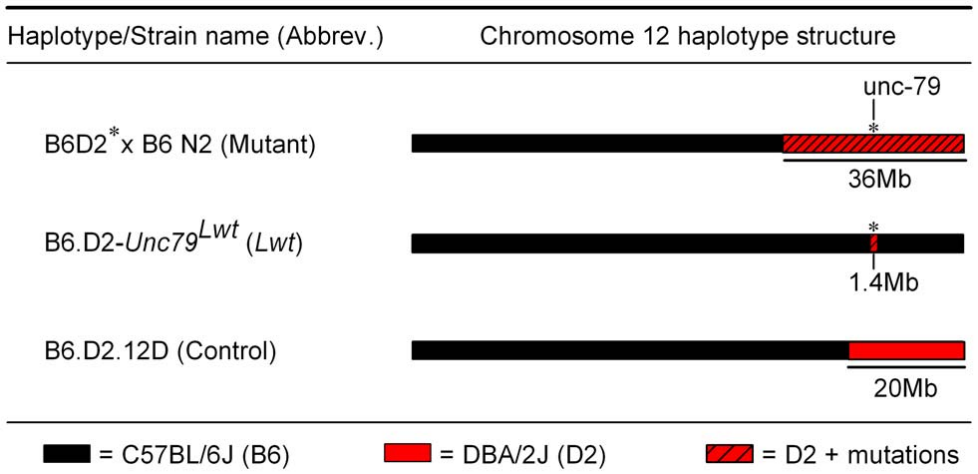
Chromosome 12 haplotype structure of animals used for behavioral testing. B6:D2*×B6 N2 (Mutant) mice harbor the mutagenized D2 chromosome 12 across the 95% confidence interval (between D12Mit158 and D12Mit263). The rest of the genome is a mixed B6 and D2 genetic background. *Lwt* and Control (see [30]) mice are congenic strains on a pure B6 genetic background. *Lwt*/+ and Control/+ males were backcrossed to either B6 or D2 females to produce populations for behavioral testing. B6 chromosomal DNA is represented in black, D2 chromosomal DNA represented in red, and mutagenized D2 chromosomal DNA is represented by hatched red.

During the course of a mouse N-ethyl-N-nitrosourea (ENU) forward mutagenesis screen designed to identify enhancers of dopaminergic neurotransmission using a sensitized genetic background, we mapped a dominant mutation that increased locomotor activity in a quantitative fashion [23]. This mutation appeared to act *independently* of the sensitized genetic background [heterozygosity for a null mutation in the dopamine transporter [23]]. The full details of the screen, including the genomewide mapping of this locus, have been described elsewhere in [23]. Briefly, we induced mutations onto the DBA/2J (D2) strain using ENU (*) and crossed these mutagenized animals to C57BL/6J (B6) mice to create B6D2^*^ F1 animals that were screened for increased locomotor activity when placed into a novel environment. One of these hyperactive B6D2^*^ animals, male 28C4, was backcrossed repeatedly to B6 females yielding a population for Quantitative Trait Locus (QTL) analysis. This analysis suggested that a dominant mutation on the D2 strain on distal mouse chromosome 12 was responsible for increased locomotor response to novelty (Figure 1A). As part of the phenotypic screening, B6D2^*^×B6 animals were weighed between the ages of 8 to 10 weeks, revealing a highly significant QTL for body weight in approximately the same location on distal mouse chromosome 12 (Figure 1B). Haplotype analysis indicated that heterozygous carriers (Mutant/+) of the distal 36.0 megabases (Mb) of the mutagenized chromosome 12 (between markers D12Mit158 and D12Mit263, see Figure 1A and Figure 2) were mildly hyperactive (Figure 1C) and weighed less (Figure 1D) than wild-type non-carriers.

**Figure 3:**
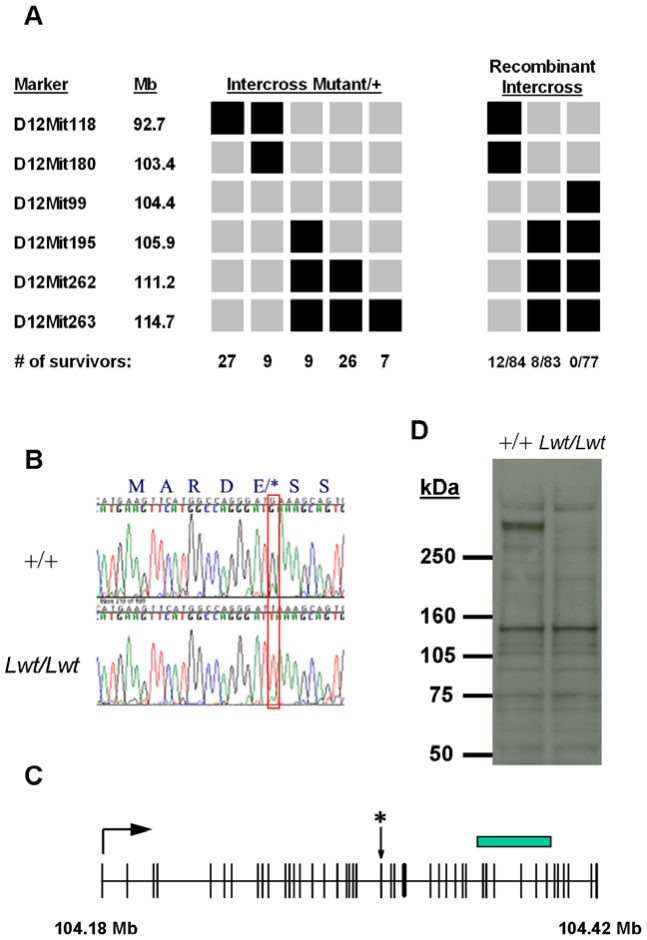
Positional cloning of the unc-79 *Lightweight* mutation. (A) Haplotype analysis of surviving animals from Mutant/+ intercross. Mutant/+ animals were intercrossed (Left plot) and animals that survived were genotyped across the distal portion of mouse chromosome 12. Black boxes indicate homozygosity for the mutagenized D2 strain and grey boxes indicate either homozygosity for the B6 strain or heterozygosity for B6 and D2. Numbers of surviving animals with a particular haplotype are indicated at the bottom. Three different male carriers (Right plot) with the indicated haplotypes were crossed to Mutant/+ females, and the ratio of surviving progeny/total number born was monitored. Both crosses indicate that the lethality maps to a region between D12Mit180 and D112Mit195. Note that survival of animals born to Mutant/+ females is lower than expected for unknown reasons. (B) Sequence analysis of +/+ and *Lwt*/*Lwt* DNA. Note the G→T transversion in *Lwt/Lwt* which changes a glutamic acid residue to a premature stop codon. (C) Exon structure of *unc-79* gene (AB257853) with position of *Lwt* point mutation indicated by (*). Genome coordinates were determined using NCBI Build 37. The C-terminal portion of the protein used to generate the antibody is indicated by a rectangle. (D) Western analysis of +/+ and *Lwt*/*Lwt* P0 whole brain lysates. Note the absence of unc-79 protein in *Lwt/Lwt* homozygotes at ∼300 kDa.

When Mutant/+ animals were intercrossed, progeny that were homozygous for the mutagenized chromosome across the 95% confidence interval did not survive beyond postnatal day one (P1), despite the fact that homozygous mutants were phenotypically indistinguishable from wild-type pups at birth and were born at the expected Mendelian ratios. We hypothesized that the same mutation was responsible for the locomotor, weight and lethal phenotypes and used the lethal phenotype to fine map the mutation. We intercrossed mutant carriers extensively and genotyped animals that survived to adulthood. Haplotype analysis indicated that lethality mapped to a small region between the markers D12Mit180 and D12Mit195 (Figure 3A, left panel). We confirmed this observation by crossing three different males with recombination events surrounding these markers to Mutant/+ females. Analysis of survivors indicated that lethality was indeed caused by a mutation within this region (Figure 3A, right panel). Additional single nucleotide polymorphisms narrowed the non-recombinant interval to ∼1.5 Mb. Exon sequencing of seven candidate genes within this region revealed a single nonsense point mutation in the mouse homolog of the *C. elegans unc-79* gene. The mutation results in a G→T transversion at amino acid position 1292 leading to the conversion of a glutamic acid residue to a premature stop codon (Figure 3B) in exon 27. This change was not found in either the B6 or D2 wild-type strains. Given the reduced body weight and the subsequent discovery (described below) that these mice are hypersensitive to the sedative effects of ethanol, we have assigned the allele name *Lightweight* to this mutation.

Northern blot and *in situ* hybridizations indicate that the *unc-79* mRNA is widely expressed throughout the central nervous system, and as expected there is a modest reduction in mRNA in homozygous mutant P0 brain tissue, presumably due to nonsense mediated decay (Figure S1). To determine whether a full-length unc-79 protein was produced in mutants, we generated polyclonal antibodies against a C-terminal portion of the protein (see Figure 3C). Immunoblot analysis of P0 whole brain tissue lysates from wild-type and homozygous mutant animals indicates an absence of the expected band at ∼290 kDa in mutant versus wild-type homogenates (Figure 3D). In invertebrates, null alleles of the unc-79 gene dramatically influence expression of the NALCN protein homolog [10]–[12]. Western blot analysis of NALCN expression in postnatal day 0 (P0) wild-type and *Lwt/Lwt* littermates did not reveal any obvious differences in expression (Figure S2).

### Lethality, locomotor, and weight phenotypes are captured in *Lightweight* congenic mice

The quantitative increase in locomotor activity and decreased body weight of *Lightweight* mutants could arise from or be influenced by other ENU-induced point mutations or polymorphic differences between the B6 and D2 strains. Thus, several experiments were performed to further explore these possibilities. First, a ∼1.4 Mb region surrounding the *Lightweight* mutation was introgressed fully onto the C57BL/6J genetic background to create the B6.D2-*Unc79^Lwt^* (*Lwt*) congenic (see Figure 2). We confirmed the uniformity of the background by genotyping against a panel of 555 informative SNPs spaced evenly across the genome [24].

The mutation rate of ENU depends on many factors, but hovers around 1×10^−6^ [25]–[27]; therefore, it is unlikely that more than one or two point mutations were induced within this congenic region, and furthermore, the likelihood of mutating a coding exon is very rare [28]. Regardless, sequencing of the majority of coding exons in this congenic interval did not reveal any additional mutations other than *Lightweight*. Importantly, intercrosses of *Lightweight* heterozygous animals have confirmed that the homozygous lethality is contained within this congenic interval (D.J.S. and D.C., data not shown). These data are consistent with the previous observation that a homozygous knockout allele of the *unc-79* gene is perinatal lethal [29].

**Figure 4:**
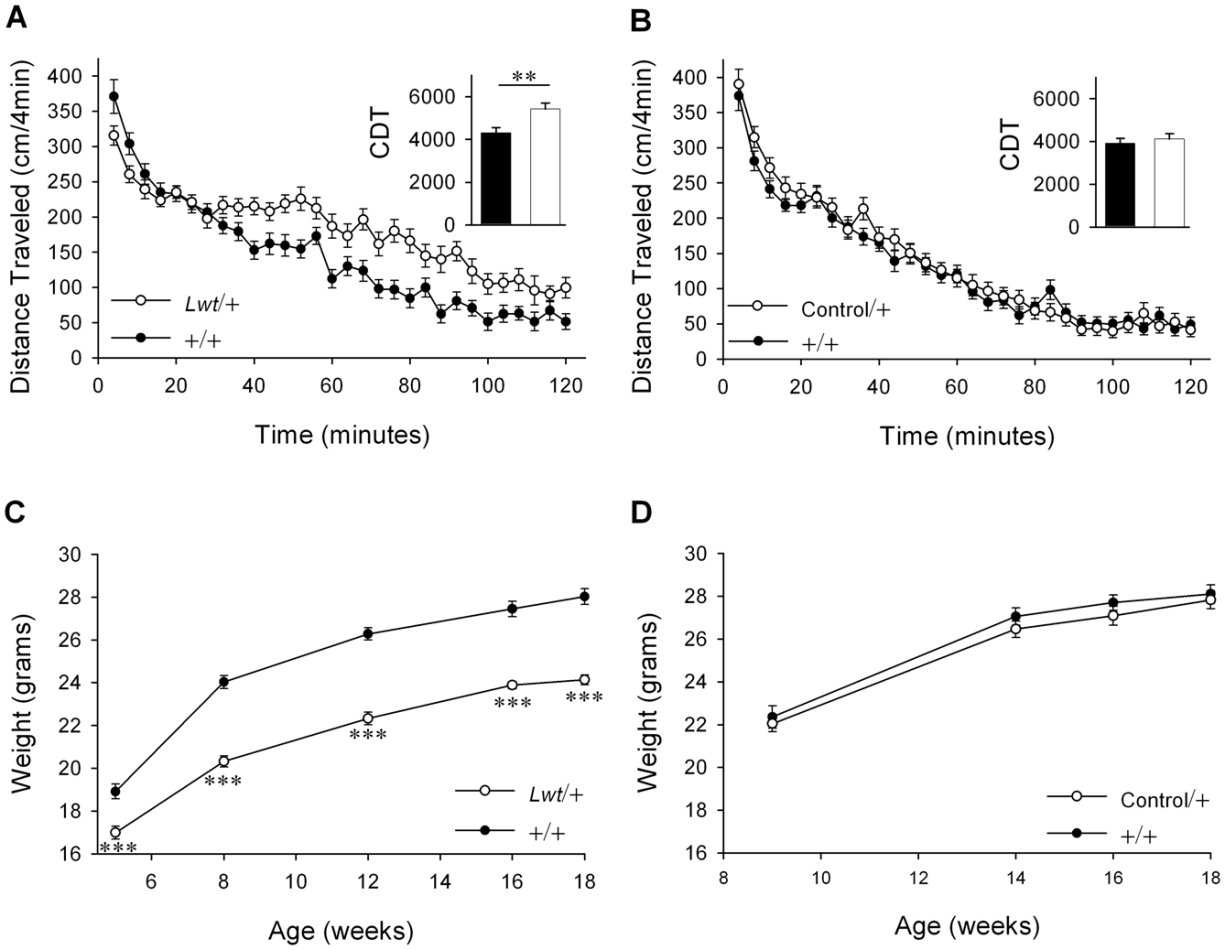
B6.D2-*Unc-79^Lwt^* (*Lwt*) congenic strain captures locomotor and body weight phenotypes. (A) Spontaneous locomotor activity was assayed in +/+ (n = 34) and *Lwt*/+ (n = 26) male littermates. *Inset:* Cumulative distance traveled (CDT) over two hours (in centimeters) in *Lwt*/+ males (white bar) was significantly higher than in +/+ (black bar) littermates (**P<0.01, Student's t-test). (B) Congenic strain B6.D2.12D heterozygotes (denoted here and elsewhere as Control/+) was used to control for polymorphic differences between the B6 and D2 strains on mouse distal chromosome 12. There was no significant difference between +/+ (n = 34) and Control/+ (n = 34) male littermates in locomotor activity. *Inset:* Cumulative distance traveled (CDT) over two hours (in centimeters) did not differ significantly between Control/+ (white bar) and +/+ (black bar) male littermates (Student's t-test). (C) At all ages tested *Lwt*/+ (n = 13) male animals weighed less than their +/+ (n = 15) littermates (***P<0.001, *post hoc* Tukey test). (D) Control/+ (n = 15) and +/+ (n = 15) male littermates did not differ significantly in body weight.

Using this congenic strain, *Lightweight* heterozygotes were confirmed to be modestly hyperactive (Figure 4A inset, *P*<0.01, Student's t-test) and to weigh less than their wild-type littermates [F_1,104_(genotype) = 87.0, *P*<0.001] (Figure 4C). To rule out the possibility that a polymorphism in the D2 strain influences these phenotypes, the congenic B6.D2.12D (“Control”) strain (see Figure 2) [30] was also examined. This non-mutagenized, congenic strain harbors a distal portion of the D2 chromosome 12 encompassing all of the genes introgressed in the *Lightweight* congenic, controlling for all strain-specific polymorphisms in *Lightweight* animals. Importantly, no difference was observed between Control/+ mice and their wild-type littermates in locomotor activity (*P* = 0.75, Student's t-test) (Figure 4B) or weight [F_1,56_(genotype) = 0.45, *P* = 0.51] (Figure 4D). Taken together, these data strongly support the conclusion that the *unc-79* mutation, and not some other lesion or polymorphism, is responsible for the observed phenotypes.

### *Lightweight* heterozygotes exhibit subtle alterations in body composition and food consumption

Because *Lwt*/+ animals weigh less than wild types, food and water consumption was monitored for 19 days beginning at approximately postnatal day 80 (P80) and followed by body composition analysis at P100. Analysis of age adjusted data (Model 1, see Materials and Methods) indicate that *Lwt*/+ mice are shorter in length and weigh less than +/+ mice. In addition, *Lwt*/+ mice consume similar amounts of food and water, yet accumulate less fat than +/+ mice (Table 1).

**Table 1:** Body composition and food and water consumption of male +/+ and *Lwt*/+ mice (value ± SEM).

Measure	Model	+/+ (n = 16)	*Lwt*/+ (n = 16)	*P* value
**Sacrifice weight (SACW) (g)**		26.2±0.34	23.3±0.34	**<0.0001**
**Total food intake (g)**	Model 1	27.75±0.59	27.50±0.59	N.S.
	Model 2	25.87±0.53	29.38±0.53	**0.0005**
**Total water intake (ml)**	Model 1	28.4±1.1	26.6±1.1	N.S.
	Model 2	25.6±1.1	29.5±1.1	0.052
**ECW (g)**	Model 1	10.092±0.156	9.943±0.156	**0.0002**
	Model 2	10.279±0.068	10.579±0.068	**0.0155**
**Total fat (mg)**	Model 1	907±28	645±28	**<0.0001**
	Model 2	826±27	727±27	**0.0395**
**Nasal-anal length (mm)**	Model 1	93.5±0.6	90.5±0.6	**0.0017**
	Model 2	93.1±0.8	90.8±0.8	0.0856

Model 1: unadjusted raw data. Model 2: sacrifice weight included as covariate.

ECW, empty carcass weight (a measure of lean body mass).

To determine if differences in food and water intake, NA (nasal-anal) length, empty carcass weight (ECW), and total fat (TF) were due to the size of the animal, we re-analyzed the phenotypic data to include sacrifice weight (SACW) as a covariate (Model 2, see Materials and Methods). These results indicate that if *Lwt*/+ mice weighed the same as +/+ mice they would consume significantly more food on a gram per kilogram basis. This analysis also showed that the carcass of *Lwt*/+ mice is heavier and reduced in total fat. Thus, *Lwt*/+ mice have a larger proportion of body weight as lean mass and a smaller proportion as body fat (Table 1).

### *Lightweight* heterozygotes have altered acute responses to ethanol and anesthetics

**Figure 5:**
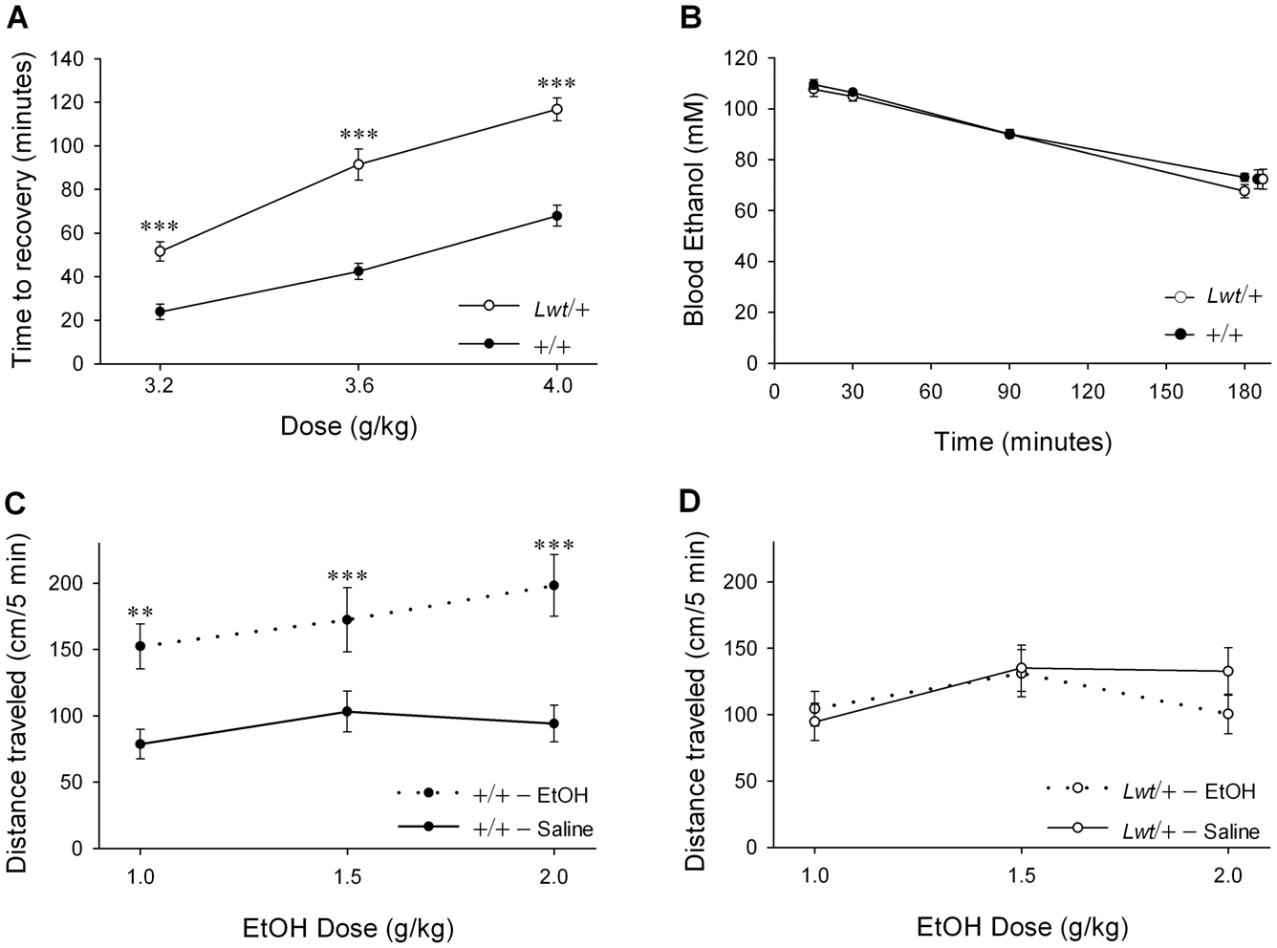
Alterations in acute responses to ethanol in *Lwt*/+ congenic mice. (A) Duration of loss of righting reflex in response to the indicated dose of ethanol (g/kg, i.p.) was determined for both *Lwt*/+ (n = 13–18) and +/+ male littermates (n = 12–23). There was a highly significant increase in time to recovery at all doses tested in *Lwt*/+ animals (***P<0.001, *post hoc* Tukey test). (B) Male *Lwt*/+ (n = 9) and +/+ littermates (n = 9) were tested for ethanol clearance after a 3.6 g/kg injection of ethanol (i.p.). These animals were sampled repeatedly at the times indicated. There were no significant differences between the genotypes at any timepoint. In addition, three additional animals of each genotype were sampled only at the 180 minute timepoint to control for repeated sampling. No significant differences were found between the 180 minute values from the repeatedly and acutely sampled groups. (C) Mean locomotor activity of +/+ male mice (n = 12–16) for the first five minutes after treatment with either saline (solid lines) or ethanol (dotted lines). (D) Mean locomotor activity of *Lwt*/+ male mice (n = 12–15) for the first five minutes after treatment with either saline (solid lines) or ethanol (dotted lines). Note that there is significantly higher locomotor activity (*P* = 0.038, *post hoc* Tukey test) in response to saline injection in *Lwt*/+ mice relative to +/+ mice in the locomotor activation experiment shown in (C) and (D). (**P<0.01; ***P<0.001, *post hoc* Tukey test).

*C. elegans unc-79* mutants exhibit altered responses to anesthetics and ethanol [4]–[6]. Since *unc-79* mRNA is reduced in *Lwt*/+ mice, the response of *Lightweight* heterozygotes to ethanol and inhaled anesthetics was examined. A highly significant increase in the sensitivity to the acute sedative effects of ethanol in *Lwt*/+ mice relative to +/+ littermates was observed by the loss of righting reflex test [F_1,58_(genotype) = 64.0, *P*<0.001] (Figure 5A). The B6.D2.12D congenic strain was also tested for ethanol-induced loss of righting reflex (3.6 g/kg, i.p.). Importantly, no difference was observed between the Control/+ mice and their wild-type littermates (D.J.S. and D.C., data not shown).

Because differential absorption, distribution, or clearance of ethanol may have contributed to the increased righting response time observed in *Lwt*/+ mice, blood ethanol concentrations (BEC) were measured for 180 minutes after a single administration of ethanol (3.6 g/kg, i.p.). A parallel experiment was also performed to control for longitudinal sampling, where a single blood sample was obtained from a separate cohort at t = 180 minutes. Importantly, we did not observe a difference in BEC at any timepoint between *Lwt*/+ mice and wild-type littermates [F_1,48_(genotype) = 0.63, *P* = 0.44] (Figure 5B).

Acute locomotor activation produced by ethanol was also investigated (1.0, 1.5 and 2.0 g/kg). We noted that although they had been fully habituated to the testing chambers prior to injection, *Lwt*/+ mice exhibited a slight but significant increase in locomotor activity in response to saline injection relative to +/+ littermates (*P* = 0.038, *post hoc* Tukey test). Wild-type animals exhibited significant activation at all doses tested (*P*<0.01, *post hoc* Tukey test) (Figure 5C). In contrast, no locomotor activation in response to ethanol injection was observed in *Lwt*/+ mice (Figure 5D).

**Figure 6:**
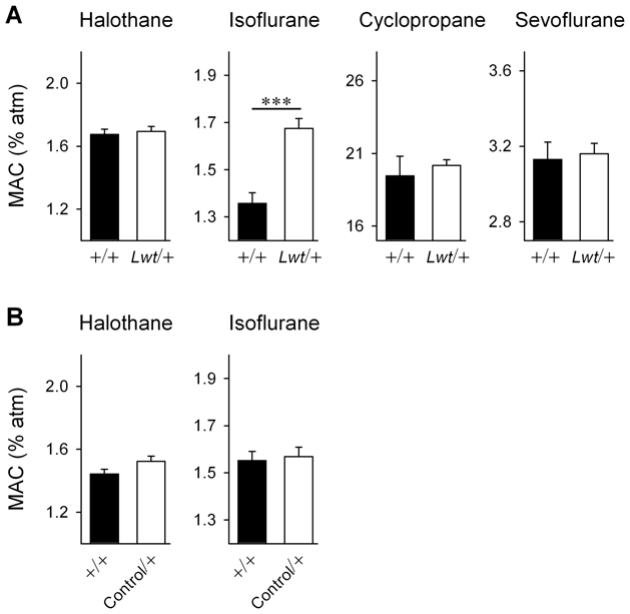
*Lwt*/+ congenic mice are *resistant* isoflurane anesthesia. (A) The minimum alveolar concentration (MAC) required to suppress movement in response to a tail pinch was determined for several anesthetic agents in both *Lwt*/+ (white bars) (n = 9) and +/+ littermates (black bars) (n = 9). There was no significant difference in MAC for halothane, cyclopropane, or sevoflurane; however, there was a significant increase in the MAC in *Lwt*/+ animals in response to isoflurane (***P<0.0001, Student's t-test). (B) MAC was determined for halothane and isoflurane in Control/+ (white bar) (n = 13) and +/+ (black bar) (n = 13) male littermates. There was no significant difference between genotypes in MAC.

The response to inhaled anesthetics was evaluated by determining the minimum alveolar concentration (MAC) of anesthetic required to suppress movement in response to a tail pinch in 50% of the mouse population. *Unc-79* worms are hypersensitive to the immobilizing effects of a various inhaled anesthetics, particularly halothane [4], [5]. We did not observe any alteration of MAC in response to halothane, cyclopropane, or sevoflurane in *Lightweight* heterozygotes. However, there was a significant *resistance* to isoflurane-induced anesthesia relative to wild-type littermates (*P*<0.0001, Student's t-test) (Figure 6A). There was no difference in response to either halothane or isoflurane in Control/+ mice when compared to their wild-type littermates (Figure 6B).

### *Lightweight* heterozygotes consume more ethanol than wild types and establish conditioned place preference to ethanol

**Figure 7:**
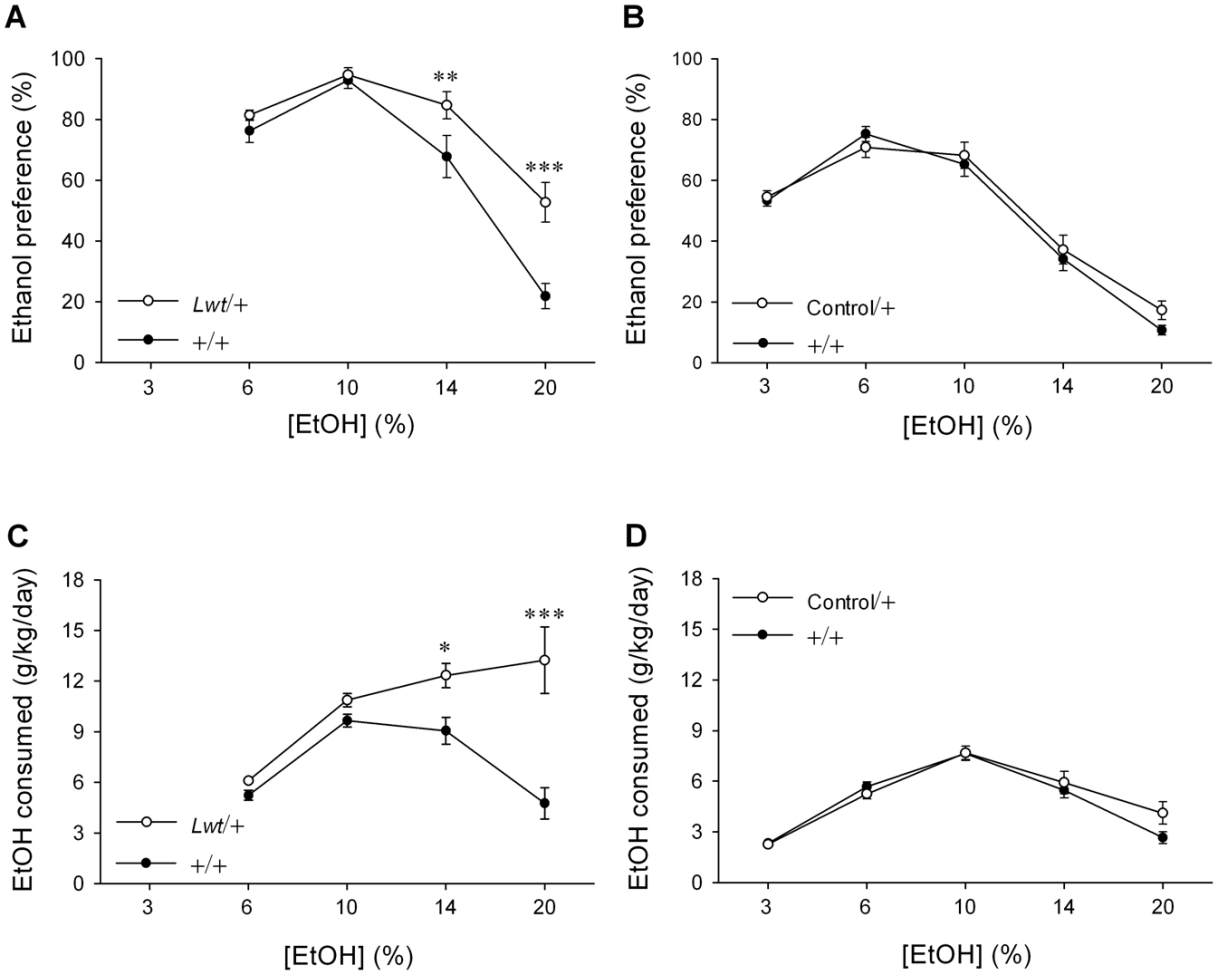
*Lwt*/+ congenic mice exhibit a higher preference for and consumption of ethanol. (A) *Lwt*/+ (n = 13) animals have a higher preference for ethanol than +/+ littermates (n = 14). (B) There was no difference in ethanol preference in Control/+ (n = 15) relative to +/+ (n = 15) littermates. (C) *Lwt*/+ (n = 13) animals consume more ethanol than +/+ littermates (n = 14). (D) There was no difference in ethanol consumption in Control/+ (n = 15) relative to +/+ (n = 15) littermates. (*P<0.05; **P<0.01; ***P<0.001, *post hoc* Tukey test).

**Figure 8:**
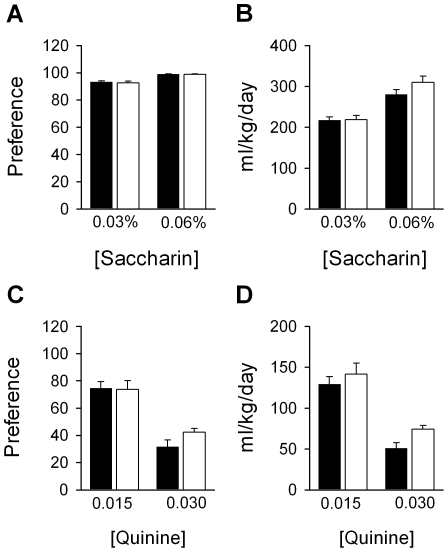
Taste preference is not altered in *Lwt*/+ congenic mice. Relative to +/+ littermates (n = 14) (black bars), *Lwt*/+ (n = 13) (white bars) animals do not show differences in preference for (A) or consumption of (B) saccharin (%, w/v), or in preference for (C) or consumption of (D) quinine (units in mM). The same is true for the control strain (data not shown).

Because *Lightweight* mice have altered acute responses to ethanol, other ethanol-related behaviors were investigated on a B6 background. First, ethanol preference and consumption was examined using a two bottle choice paradigm in which individually-housed animals were given access to water or increasing concentrations of ethanol (from 3% to 20%) *ad libitum*. Relative to +/+ mice, *Lwt*/+ animals exhibited a higher preference for ethanol [F_1,76_(genotype) = 9.9, *P* = 0.004; F_3,76_(genotype×concentration) = 6.6, *P*<0.001] and consumed more ethanol [F_1,76_(genotype) = 14.5, *P*<0.00; F_3,76_ (genotype × concentration) = 6.9, *P*<0.001]. Increased preference and consumption was particularly pronounced at the higher concentrations tested (Figure 7A and 7C). It should be noted that no ethanol preference QTLs between the B6 and D2 strains have been mapped to distal chromosome 12. This was confirmed when we determined that no differences were observed between Control/+ mice relative to +/+ mice in preference for ethanol [F_1,112_(genotype) = 0.35, *P* = 0.56] or consumption of ethanol [F_1,112_(genotype) = 0.40, *P* = 0.53] (Figure 7B and 7D). Further, there were no alterations in taste sensitivity in *Lwt*/+ mice relative to +/+ littermates for either preference [F_1,26_(genotype) = 0.23, *P* = 0.65] or consumption [F_1,26_(genotype) = 0.22, *P* = 0.64] of saccharin-containing solutions over water (Figure 8A and 8B) or preference [F_1,26_(genotype) = 0.74, *P* = 0.40] or consumption [F_1,26_(genotype) = 2.1, *P* = 0.16] of quinine-containing solutions over water (Figure 8C and 8D). Additionally, no differences in taste sensitivity were observed in the control strain (D.C. and D.J.S., data not shown).

The influence of the *Lightweight* mutation on ethanol preference and consumption was also investigated on a B6D2 F1 hybrid genetic background. In general, ethanol consumption was much lower on this genetic background versus C57BL/6J (Figure S3). Significant increases in both preference and consumption were observed at the 6% and 10% ethanol concentrations (*P*<0.01, *post hoc* Tukey test). When the congenic Control/+ mice were tested on a B6D2 F1 genetic background, no alterations were observed in ethanol preference or consumption (Figure S3).

It appears, however, that differences in saccharin preference and total fluid consumption may influence voluntary ethanol consumption on this background. One should note that B6D2 F1 *Lwt*/+ mice consume more water *ad libitum* than +/+ animals (*P* = 0.025, Mann-Whitney Rank Sum Test). While *Lwt*/+ mice did not exhibit alterations in preference for either 0.03% or 0.06% saccharin or quinine-containing solutions, they did exhibit increased consumption of saccharin relative to +/+ animals (Figure S4). This result is reflected in a greater total fluid consumption (water+saccharin solution) in *Lwt*/+ mice relative to +/+ animals (Figure S4). Because saccharin preference is very high in both genotypes (nearing 100%), it is possible that this ceiling may mask a difference in saccharin preference. For this reason, the taste preference test was repeated on a separate set of B6D2 animals using a lower saccharin concentration (0.015%) from which an increased saccharin preference was observed in *Lwt*/+ mice relative to +/+ animals (*P*<0.001, *post hoc* Tukey test) (Figure S4). We also observed increased total fluid consumption (water + quinine solution) in *Lwt*/+ animals. In contrast, there were no significant differences in taste sensitivity or in total fluid consumption in Control/+ B6D2 F1 animals (D.J.S., data not shown). Together, these data suggest that while ethanol preference and consumption are indeed increased in *Lwt*/+ mice on a B6D2 background, both increased fluid consumption and a higher propensity to prefer saccharin may contribute to this phenotype.

**Figure 9:**
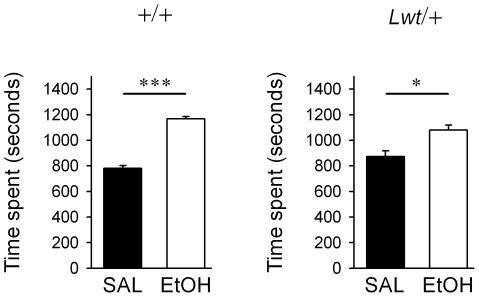
*Lwt*/+ congenic mice develop a conditioned place preference to ethanol. Wild-type (n = 11) and *Lwt*/+ (n = 11) littermates developed a conditioned place preference to a 1.75 g/kg dose of ethanol. (*P<0.05; ***P<0.001 compared with saline).

Lastly, the propensity of *Lwt*/+ mice to express a conditioned place preference (CPP) to ethanol (1.75g/kg, i.p.) was assessed. Wild-type animals showed a robust place preference (*P*<0.001, Wilcoxon signed rank test), while *Lwt*/+ mice develop a significant place preference (*P* = 0.032, Wilcoxon signed rank test) (Figure 9). There is not a significant difference between the two genotypes in time spent on the ethanol-conditioned side (*P* = 0.0818, Mann-Whitney test) (Figure 9), although there is a trend towards greater ethanol CPP in wild type animals. We tested 11 animals per genotype; it is possible that by testing larger numbers of animals, we would detect a significant difference between *Lwt*/+ and +/+ animals.

### Hypersensitivity to ethanol in *C. elegans unc-79*, *unc-80*, and *nca-1;nca-2* double mutants suggests a conserved role of these genes in ethanol-related behaviors

**Figure 10:**
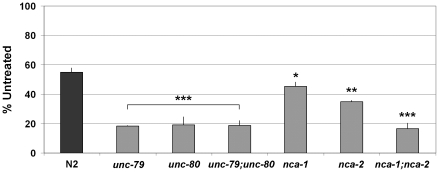
Swimming behavior is hypersensitive to ethanol in *C. elegans unc-79*, *unc-80*, and *nca-1;nca-2* double mutants. Swimming sensitivity to ethanol (400mM). The mean relative frequency of body bends during swimming of wild-type (N2) and various mutants is shown. Error bars indicate SEM. Asterisk indicates a statistical difference as tested by Student's t-test (*P<0.05, **P<0.005, ***P<0.0001).

Our observations in *Lwt*/+ mice led us to revisit the possibility that *C. elegans unc-79* mutants were also hypersensitive to acute ethanol exposure. A previous study reported that *unc-79* mutants were modestly resistant to ethanol-induced immobility in liquid media [6]. They also reported no alteration in sensitivity to ethanol in *unc-80* mutants [6]. In contrast, we observed a pronounced hypersensitivity to ethanol in *unc-79*, *unc-80*, and *nca-1;nca-2* double mutants when compared to wild-type N2 animals (Figure 10).

## Discussion

We have identified and cloned a point mutation in the mouse homolog of the *unc-79* gene, to which the allele name *Lightweight* was assigned due to the resultant phenotypic traits. The *Lightweight* mutation creates a premature stop codon in the middle of the unc-79 protein. We introgressed a ∼1.4 Mb region surrounding the *Lightweight* mutation fully onto the C57BL/6J genetic background. *Lwt/Lwt* homozogotes are born at the expected Mendelian ratios and appear normal at birth, but survive a maximum of two days, similar to what has been reported for a knockout allele of this gene [29]. Although *Lightweight* heterozygotes exhibit a normal birthweight, soon thereafter and as adults, body weight is significantly less than their wild-type littermates. In addition, *Lightweight* heterozygotes have increased lean tissue and reduced body fat in comparison to wild-type littermates. Furthermore, *Lightweight* heterozygotes consume more food on a B6 background and more water on a B6D2 F1 background. Together, these results suggest a higher metabolic rate, increased protein turnover, or differences in energy usage.

By testing a control congenic strain in parallel, we have demonstrated that strain-specific polymorphisms from the introgressed D2 background do not produce or influence any of the phenotypes observed here. This possibility is often ignored [31] but is an important concern due to the quantitative nature of several of the phenotypes tested. Although we have ruled out strain-specific polymorphisms, we cannot formally rule out the possibility that an additional ENU-induced mutation in our congenic strain might influence some of the phenotypes we have observed. The mutation rate of ENU depends on many factors, but hovers around 1×10^−6^ [25]–[27]; therefore, it is unlikely that more than one or two point mutations were induced within this 1.4 Mb congenic region, and furthermore, the likelihood of mutating a coding exon is very rare [28]. We have sequenced the majority of coding exons in this congenic interval without finding any additional mutations other than *Lightweight*. Complete resequencing of the whole 1.4 Mb interval would confirm the absence of other confounding mutations.

The alterations that we observed in *Lightweight* heterozygotes in response to ethanol and anesthetics are qualitatively similar to what has been reported previously in *C. elegans* and *Drosophila*; however, important differences exist. In invertebrates, *unc-79* mutants are very hypersensitive to halothane [10]; whereas, we observed no alterations in MAC for halothane in *Lightweight* heterozygotes. However, we did observe that *Lightweight* heterozgotes are *resistant* to isoflurane. It is unclear at this point why these species differences exist. One possibility is that most of the *unc-79* alleles tested in invertebrates were true null mutations; whereas, we tested heterozygous mutant mice. Another possibility is that a truncated protein might be produced in *Lightweight* heterozygotes, which could interfere with the proper functioning of this pathway in unanticipated, even opposite, ways. It should be noted that the fact that *Lightweight* heterozygotes were resistant to isoflurane and not to all anesthetic agents tested suggests that the anesthetic phenotype does not simply reflect a non-specific alteration in neuronal function.

The finding that *C. elegans unc-79*, *unc-80*, and *nca-1;nca-2* double mutants were hypersensitive to acute ethanol exposure suggests that this pathway is important for determining ethanol sensitivity. As in vertebrates, UNC-79 and UNC-80 proteins are thought to regulate the NALCN-related leak channel encoded by *nca-1* and *nca-2* [32]. It was possible however that the ethanol phenotypes of *unc-79* and *unc-80* were due to additional, as yet undiscovered, roles of these genes. The fact that the *nca-1;nca-2* double mutant exhibits the same ethanol hypersensitivity suggests that the normal function of this leak channel is indeed required for ethanol responses. Ethanol may directly modulate the nca-encoded leak channel or perhaps indirectly modulate the channel through an effect on *unc-79* and *unc-80*. The ethanol hypersensitivity of *Lightweight* mice may also reflect defective NALCN function.

Because *Lightweight* animals exhibited dramatically altered responses to acute ethanol injection, we assayed free choice ethanol preference and consumption using a two bottle choice test. On both a B6 and a B6D2 F1 hybrid background, *Lightweight* heterozygotes displayed a higher preference for and consumption of ethanol than wild-type littermates. However, on a B6D2 F1 background, in addition to having a higher preference for and consumption of ethanol (at 6% and 10%), *Lightweight* animals also consume more water than wild-type littermates, and when offered water or water plus a tastant (saccharin), they tend to choose the bottle containing the tastant over water alone. Given these observations, in combination with the body composition and food consumption analysis performed in B6 *Lightweight* animals, we favor a conservative interpretation of the two bottle choice data: *Lightweight* animals have a higher preference for and consumption of ethanol; however, we cannot exclude the possibility that taste preference and/or alterations in metabolism could contribute to this phenotype. Because the unc-79 protein is widely expressed throughout the central nervous system, tissue-specific deletion using conditional mutagenesis or RNA interference may yield more specific results. Nonetheless, it is clear that *Lightweight* heterozygotes voluntarily consume more ethanol than wild-type littermates.

There is a consistent correlation between rodent populations that voluntarily consume more ethanol in a two bottle choice assay and increased operant oral self administration of ethanol [33]. Because oral self administration of ethanol stimulates dopamine release [34], it is possible that the unc-79 gene product acts in the ventral tegmental area (VTA) to mediate or modulate NALCN function. In dopaminergic VTA neurons, neurotensin and other neuromodulators activate a nonselective cationic conductance with properties similar to the NALCN channel [18]. Furthermore, a neurotensin- and substance P-activated inward sodium current is absent from cultured NALCN knockout VTA neurons [13]. It is possible that ethanol acts directly upon NALCN channels in the VTA to influence firing rate. Alternatively, ethanol might alter the release of neurotensin, substance P, or other neuromodulators in the VTA to influence NALCN/unc-79 function secondarily. Testing of these hypotheses in the future may lead to insight into the mechanism of action of ethanol on the nervous system and specifically into mechanisms of reward-related behavior.

In conclusion, the conservation of ethanol and anesthesia phenotypes across species suggests that *unc-79* and associated *unc-80* and NALCN genes are part of a novel and relatively unexplored pathway that mediates the physiological effects of these agents. It is notable that we have observed a wide variety of phenotypes in heterozygotes. If variants of *unc-79* and associated genes exist in the human population, it is possible that even heterozygotes may exhibit ethanol- and weight-related phenotypes.

## Materials and Methods

### Animals

All animal use was approved by the institutional animal care and use committee of the Gallo Center in accord with the guidelines for animal use laid out by the US Public Health Service. Unless stated otherwise, all animals were group-housed in a climate controlled facility on a 12 hr light/dark cycle (lights on at 07:00), and food (Picolab Rodent Diet 20 #5053, Lab Diet) and water were available *ad libitum*. All experimentation was carried out at the same time of the day by the same investigator. Only male animals were used for behavioral experiments.

The congenic strain B6.D2-*Unc79^Lwt^* harboring the *Lightweight* mutation was produced by backcrossing an ∼1.4 Mb region of mutagenized D2 genome from chromosome 12 onto the C57BL/6J (B6) strain for >10 generations. A genetically uniform B6 background was confirmed by genotyping this strain with a panel of 555 informative SNPs [24]. To produce animals for behavioral testing, B6.D2-*Unc79^Lwt^* heterozygous males were backcrossed to C57BL/6J females, with the exception of determination of MAC (the Minimum Alveolar Concentration of anesthetic required to abolish a tail pinch response in 50% of animals) and one of the ethanol preference tests, where B6.D2-*Unc79^Lwt^* heterozygous males were backcrossed to DBA/2J females.

A non-mutagenized congenic strain, B6.D2.12D (“Control”), was obtained from UCLA and used to control for strain specific polymorphisms on distal chromosome 12 [30].

### ENU mutagenesis and QTL mapping

Detailed methods used for the screen have been reported previously [23]. Briefly, adult DBA/2J (D2) male mice (Jackson Labs) were administered three doses of 90 mg/kg N-ethyl-N-nitrosourea (ENU, indicated by *) (Sigma) intraperitoneally at weekly intervals as described [35]. Approximately 12 weeks after the last injection, these mice recovered fertility and were bred to non-mutagenized D2 female mice to produce G1 mutagenized male mice. These mutated D2^*^ males were then backcrossed to B6 females to produce B6D2* G2 animals that were screened for increased locomotor activity. High-scoring animals were then backcrossed repeatedly to B6 females for heritability testing and QTL mapping.

QTL analysis was performed using R/qtl (www.r-project.org/) [36]. Single loci associated with the traits were detected by interval mapping [37]. Significance thresholds for genome scans were determined according to [38]. Across chromosomal regions where all individuals were genotyped (including chromosome 12), significance thresholds were determined by permutation testing [39].

### Locomotor activity

On the testing day, male mice were transported to the testing room between the hours of 0900 and 1100 and were habituated to the room for two hours. Locomotor activity was assayed using infrared activity monitors (Accuscan, Columbus, OH) surrounding an open field of 8×8×11 inches. Distance traveled was monitored for a period of two hours. Two acrylic boxes fit inside a single Accuscan monitor, enabling us to test two animals simultaneously. Whether another animal is present in the second box affects locomotor behavior in this assay (D.S. and A.S.P, unpublished observations). We found that testing two animals from different home cages in a single Accuscan monitor both reduced variability and allowed high-throughput rates. Activity monitors were themselves housed inside sound-attenuating chambers (Med-Associates) equipped with lights and fans, both of which were turned on during the testing session. Acrylic boxes were rinsed with hot water and dried and then wiped down with a solution of 2.5% glacial acetic acid between testing sessions.

### Sequence analysis

Primers flanking the exons of candidate genes were used to amplify genomic DNA from wild-type and homozygous mutant animals. Amplicons were fluorescently labeled and sequenced on an ABI 3700 capillary sequencer (ABI, Foster City, CA). Sequence data was processed using Mutation Surveyor software (Softgenetics, State College, PA).

### Antibody generation and western analysis

A fragment encoding 327 amino acids (1585–1912 in AB257853) of the unc-79 protein was subcloned into the pGEX-4T1 vector (GE Healthcare). The resulting GST-fusion protein was purified and used as an antigen to raise chicken polyclonal antibodies (Aves Antibodies, Tigard, OR). A MBP-fusion to the same 327 amino acid stretch was used for subsequent affinity purification. Whole brain lysates from wild-type and *Lwt/Lwt* mutant P0 animals were electrophoresed on 4–12% reducing Tris-Acetate gels and transferred to a nitrocellulose membrane (Invitrogen, Carlsbad, CA). After incubation with primary antibody, a goat anti-chicken HRP-conjugated secondary antibody (Aves Antibodies) and ECL reagent (GE Healthcare) was used for detection.

### Analysis of body composition and food and water consumption

A total of 32 B6 male mice (16 +/+ and 16 *Lwt*/+) were weighed to the nearest 0.1 g every 7 days from 50 to 100 days of age. Sacrifice weight (SACW) was determined at 100±4 days, approximately 12 hours prior to dissection. Beginning on day 85, food and water intake were measured at 3 day intervals until sacrifice. At sacrifice, mice were anesthetized with isoflurane until the toe-pinch withdraw reflex and corneal reflex was lost and then placed over a grid without stretching to measure body lengths. Nasal anal (NA) and nasal tail (NT) lengths were measured to the nearest 1 mm by measuring the length from the tip of the nose to the base of the tail and to the end of the tail, respectively. Then the femoral, gonadal, mesenteric and retroperitoneal fat pads, and major organs (liver, spleen, kidney and testis) were harvested and weighed immediately. Empty carcass weight (ECW) (skinned carcass without organs, fat, nor tail) was also determined. Femurs were then dissected and measured to the nearest 0.1 mm with a Vernier scale. Total fat (TF) was calculated as the sum of all fat pads.

To determine the genotypic effects of the *Lwt*/+ mutation, statistical analyses were performed on all body weights, NA, NT and femur lengths, organ weights, TF, and food and water intakes by fitting two linear models. The first model accounted for the effects of genotype (*G_i_*) and only included age at sacrifice as a covariate (the range of age at sacrifice was 14 days): *y_ijl_* = *a(G_i_)* + *Age_j_* + *e_ijkl_* [Model 1]. The second model accounted for genotype and used age and SACW as covariates: *y_ijkl_* = *a(G_i_)* + *Age_j_* + *SACW_k_* + *e_ijkl_* [Model 2] to determine if changes in body length and body composition were simply due to the size of the mouse.

### Loss of righting reflex

All testing was performed on animals between the ages of 9 to 14 weeks. Mice were tested for loss of righting reflex as a function of dose following injections of ethanol (3.2, 3.6 and 4.0 g/kg, i.p.). The same animal was tested at all three doses, separated by two weeks between each test. Dose order was counterbalanced. After injection, animals were intermittently placed on their backs and tested for loss of righting reflex. Loss of righting reflex was defined as the inability to right itself three times within a 30 second interval. Duration was defined as the time interval between the loss and return of the righting reflex.

### Ethanol clearance

Mice were tested for ethanol clearance by procedures adapted from [40] and described previously [41], [42] with a 0.01 mM limit of detection. Animals were habituated to the testing room for two hours. At Time 0, animals were injected with ethanol (3.6g/kg, i.p.). Approximately 20 µl retro-orbital sinus blood samples were taken at 15, 30, 90 and 180 minutes after ethanol injection from nine animals per genotype. In addition, three animals per genotype had a single blood sample taken at 180 minutes post-injection, to control for blood volume loss that could result from repeated blood sampling. Alternating eyes were sampled to minimize trauma and animals were euthanized following the procedure. Samples were centrifuged at 4°C and plasma blood ethanol concentraions determined using gas chromatography (5890A GC, Hewlett Packard). Briefly, 7 µl serum was sealed in a GC autosampler vial (National Scientific, Rockwood, TN) with 7 µl 0.05% n-propanol, as an external pippeting standard. Samples, in duplicate, were heated to 65°C for 20 min, agitated for 30 sec, and allowed to settle for 1 min prior to pressurizing for headspace extraction into a 2 ml, depolarized loop (Tekmar Control Systems). Samples were immediately passed through a 220°C deactivated, glass lined inlet (Hewlett-Packard) and subjected to gas chromatography (He, 5 kPa) on a megabore 30 m, 1 µm film innowax column (Agilent Technologies) at a 45°C isotherm and quantified via flame ionization at 310°C in a deactivated jet and detector (Agilent Technologies). The column was purged after each sample by holding at 210°C for 1.5 min prior to cooling to 45°C over 5 min. The alcohol area under the curve (AUC) was divided by the external n-propanol standard AUC and compared to known standards from 300 mM–0.003 mM (run in duplicate, r^2^ typically = 1.00) that were freshly prepared for every run.

### Ethanol and taste-preference testing

A two-bottle choice protocol was used to measure oral self administration of ethanol. Two different genetic backgrounds were tested: B6 and B6D2 F1 hybrids. Eight to eleven week old animals were separated from littermates and housed individually for at least one week prior to testing, where they were presented with two drinking bottles filled with water. Mice were then given access to two bottles, one containing a solution of ethanol, saccharin, or quinine and the other containing water. Animals were given access to a single concentration of ethanol for a total of four days. Bottles and animals were weighed every two days, at which time bottle location was switched. Increasing concentrations of ethanol 3%, 6%, 10%, 14% and 20% were presented. Evaporation and leakage was measured in nearby empty cages. One week following the conclusion of the ethanol tests, the same animals were tested for alterations in taste preference by offering them access to water and saccharin (0.03% and 0.06% w/v) or water and quinine (0.015 and 0.03mM) (Sigma). Because of the significance differences in weight between +/+ and *Lwt*/+ animals, we first normalized consumption in terms of grams consumed per kilogram of mouse raised to the 2/3 power [43]. We found that the weight difference did not significantly influence interpretation of the experiments, so consumption data has been presented conventionally as g/kg/day or mls/kg/day.

### Locomotor activation

Prior to injection with saline or ethanol, animals were habituated to the testing apparatus for a period of two hours (exactly as described above for Locomotor activity). Animals were then injected with either saline or ethanol and locomotor activity was recorded for an additional 30 minutes at one minute intervals. One week later, the test was repeated, reversing the saline and ethanol injections. Injection order was counterbalanced by genotype. Separate cohorts of animals were used for each dose of ethanol (1.0, 1.5 and 2.0 g/kg) such that each animal received only a single injection of ethanol and a single injection of saline.

### Conditioned place preference

A two-chamber contextual place preference apparatus was used for ethanol conditioned place preference (CPP) (Columbus Instruments, Columbus, OH). One chamber was white and had a striated floor texture and the other chamber was black with a hatched floor texture, separated by a removable divider. The whole apparatus was housed inside a sound-attenuating chamber (MedAssociates, St. Albans, VT). Sound and light levels were normalized. Animals were between the ages of 8 to 11 weeks old. Animals were habituated to the testing area for two hours between the hours of 0900 to 1200 prior to testing. The removable divider was closed during training and open during the habituation and testing sessions. On the day prior to the commencement of training, animals were allowed 30 minutes free access to both chambers. Neither +/+ nor *Lwt*/+ males showed a baseline preference for either chamber; therefore, equal numbers of mice received the ethanol unconditioned stimulus in either chamber. Animals were tested at the same time each day (1200) and given a single injection and immediately placed into one chamber for precisely five minutes. Saline and ethanol injections were alternated until a total of six conditioning sessions were achieved for both saline and ethanol (12 conditioning days). A lower 1.75 g/kg dose of ethanol was used because of the hypersensitivity observed in *Lwt*/+ animals. On the testing day, animals were given access to both chambers for 30 minutes and time spent on each side was recorded [44].

### Determination of MAC

We determined MAC, the Minimum Alveolar Concentration of anesthetic required to abolish movement in response to a tail pinch in 50% of mice [45]. The investigators assessing movement/non-movement were blinded to the identity of the mouse. Each mouse supplied a single value for the MAC of one or more of the study anesthetics. A period of 45–55 minutes was used to provide equilibration with an initial anesthetic concentration, a concentration that equaled 60%–70% of the concentration ultimately required to suppress movement. Thus all mice initially moved in response to stimulation. The concentration was then increased in steps with equilibration times of 25 to 45 minutes depending on the solubility of the anesthetic (longer times for more soluble anesthetics). At the bracketing concentrations [highest concentration(s) permitting and lowest concentration(s) preventing movement in response to stimulation], the step size equaled 10% to 15% of the preceding value. Rectal temperature was measured to maintain core temperature between 36°C and 39°C with a heating pad.

MAC for each mouse was calculated as the average of the bracketing concentrations. We computed the mean and SD for each of 10 groups of mice defined by strain (+/+ versus *Lwt*/+) and anesthetic (cyclopropane, halothane, isoflurane, and sevoflurane). We determined the significance of differences between strains for a given anesthetic using student's t-test, applying a Bonferroni correction for multiple (four) comparisons. Thus, a value of P<0.0125 was regarded as significant for all comparisons.

### Analysis of anesthetic concentrations

Inhaled anesthetic concentrations were analyzed using gas chromatography as described previously [46]. A flame ionization detector was used to analyze all anesthetics. The chromatograph was calibrated with either primary or secondary standards.

### Statistics

Data are presented as mean ± SEM. SigmaStat software (Aspire Software International) or the GLM procedure in SAS v.9.2 (SAS Institute) was used to analyze data. In brief, data were evaluated via analysis of variance (ANOVA) followed by Tukey *post hoc* comparisons. Some data was analyzed with a Student's t-test with a Bonferroni correction for multiple comparisons. Some data was processed with non-parametric statistics when tests for normality failed.

For body composition analysis, Shapiro-Wilk and Levene's tests were used to test normality of residuals and homogeneity of variances under Models 1 and 2 for body compensation, respectively. Individual fat pad weights and total fat (TF) had heterogeneous variances between genotypes. However, this heterogeneity of the variance is the effect of the *Lwt*/+ genotype and is removed when correcting for body weight, thus raw data was used.

Significant differences between strains for a given anesthetic were evaluated via a Student's t-test, applying a Bonferroni correction for multiple (four) comparisons. Thus, a value of P<0.0125 was regarded as significant for all comparisons.

### Ethanol sensitivity in *C. elegans* mutants

Animals were maintained as described [47]. The wild-type strain was Bristol N2. The mutant strains used in this study are the following: *unc-79(e1068)*, *unc-80(eg684)*, *unc-79(e1068);unc-80(eg684)*, *nca-1(tm1851)*, *nca-2(gk5)*, *nca-1(tm1851)*; *nca-2(tm377)*.

Embryos were collected by bleaching and were grown on a standard nematode growth medium (NGM) plate at room temperature. Young adults were placed in a control M9 buffer (3 g KH_2_PO_4_, 6 g Na_2_HPO_4_, 5 g NaCl, and 1 ml of 1 M MgSO_4_ in 1.0 L dH_2_O and autoclaved) with or without 400mM ethanol solution. Swimming behavior was video-recorded for 1 min. from the 10 min. time point. Frequency of swim bends was measured for 20 sec. To minimize the influence of swimming differences on the ethanol sensitivity, we calculated a mean relative swimming (treated average bends/untreated average bends × 100).

## Supporting Information

Figure S1 Expression of *unc-79* mRNA in mutant and wild-type mice. (A) Reduced *unc-79* mRNA expression in *Lwt/Lwt* mutant mice. 5.0 µg poly A (+) RNA from wild-type adult and postnatal day 0 (P0) *Lwt/Lwt* and wild-type whole brain was probed for *unc-79* (upper portion) and β-actin (lower portion). *Unc-79* expression is lower in *Lwt/Lwt* mutants, presumably due to nonsense-mediated decay. (B) Expression of *unc-79* mRNA is restricted to nervous tissue in adult mice. An RNA blot containing poly A (+) RNA from various adult mouse tissues was hybridized sequentially with probes for *unc-79* (upper portion) and β-actin (lower portion). The position of the markers is shown on the left side of the panels. See Text S1 for Materials and Methods.
(0.48 MB TIF)

Figure S2 Western blot analysis of NALCN expression in *Lwt/Lwt* mice. Serial dilutions of postnatal day 0 (P0) brain lysates from a *Lwt/Lwt* pup and a +/+ littermate were blotted with rabbit α-NALCN polyclonal antibodies. There was no obvious alteration in expression of the NALCN protein. α-Tubulin antibodies were used as a loading control.
(0.29 MB TIF)

Figure S3 On a B6D2 F1 background, *Lwt*/+ congenic mice exhibit a higher preference for and consumption of ethanol in a two bottle choice test. (A) *Lwt*/+ (n = 19) animals have a higher preference for ethanol than +/+ littermates (n = 20) [F_1,148_(genotype) = 3.6, *P* = 0.064; F_4,148_(genotype × concentration) = 3.7, *P* = 0.007]. (B) There was no difference in ethanol preference in Control/+ (n = 16) relative to +/+ (n = 15) littermates [F_1,116_(genotype) = 0.01, *P* = 0.93; F_4,116_(genotype × concentration) = 0.26, *P* = 0.90]. (C) *Lwt*/+ (n = 19) animals consume more ethanol than +/+ littermates (n = 20) [F_1,148_(genotype) = 9.8, *P* = 0.003; F_4,148_(genotype × concentration) = 9.8, *P*<0.001]. (D) There was no difference in ethanol consumption in Control/+ (n = 16) relative to +/+ (n = 15) littermates [F_1,116_(genotype) = 0.17, *P* = 0.69; F_4,116_(genotype × concentration) = 0.97, *P* = 0.43]. (**P<0.01; ***P<0.001, *post hoc* Tukey test).
(0.30 MB TIF)

Figure S4 On a B6D2 F1 background, *Lwt*/+ congenic mice exhibit alterations in taste sensitivity and fluid consumption. (A) Relative to +/+ littermates (n = 20) (black bars), *Lwt*/+ (n = 18) (white bars) animals do not have altered preference for saccharin 0.03% or 0.06% [F_1,36_(genotype) = 0.70, *P* = 0.41] or (B) quinine (0.015mM or 0.030mM) [F_1,36_(genotype) = 0.09, *P* = 0.75]. (C) Consumption of saccharin is increased in *Lwt*/+ mice [F_1,36_(genotype) = 15.8, *P*<0.001; F_1,36_(genotype×concentration) = 8.2, *P* = 0.007]. (D) Consumption of quinine is not increased in *Lwt*/+ mice [F_1,36_(genotype) = 3.7, *P* = 0.061; F_1,36_(genotype × concentration) = 2.1, *P* = 0.155]. (E) Increased total fluid consumption contributes to the increased saccharin consumption [F_1,36_(genotype) = 20.6, *P*<0.001; F_1,36_(genotype × concentration) = 7.4, *P* = 0.010]. (F) Increased total fluid consumption is also observed for quinine-containing solutions [F_1,36_(genotype) = 11.9, *P*<0.001]. (G) A separate set of *Lwt*/+ animals (n = 12) and +/+ littermates (n = 12) were tested with a lower dose of saccharin (0.015%) and we observed a significant difference (***P<0.001, *post hoc* Tukey test) in preference at this concentration, suggesting that both the taste of saccharin and overall fluid consumption are altered in *Lwt*/+ mice on this background. (**P<0.01; ***P<0.001, *post hoc* Tukey test).
(0.35 MB TIF)

Text S1 Supporting Materials and Methods.
(0.02 MB DOC)
