# Supplementary figures and images for: Conserved Role of unc-79 in Ethanol Responses in Lightweight Mutant Mice
Source: PLoS Genet. 2010 Aug 12;6(8):e1001057. doi: 10.1371/journal.pgen.1001057 (PMC2920847; doi:10.1371/journal.pgen.1001057)

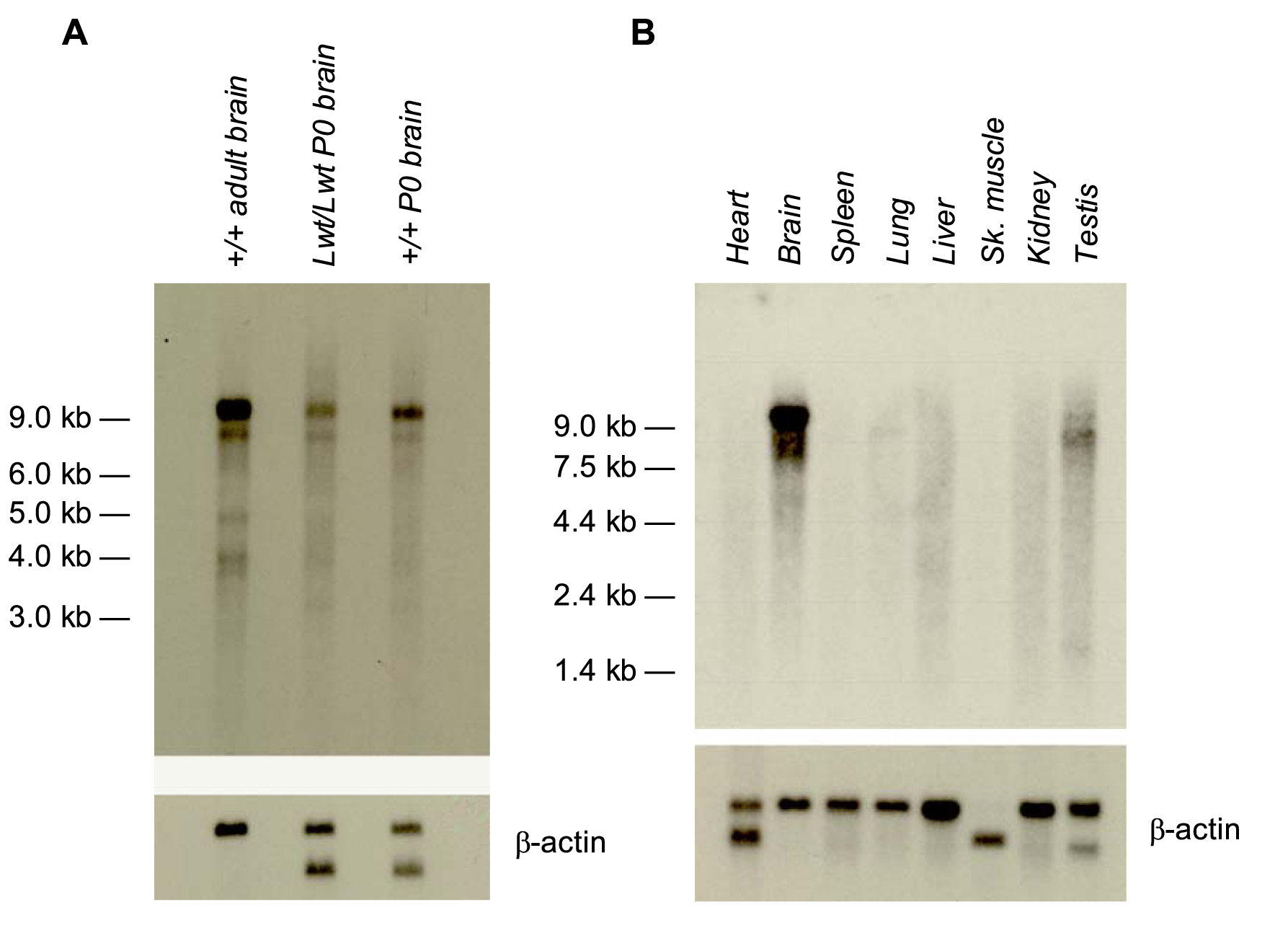

Supplement: Figure S1 — Expression of unc-79 mRNA in mutant and wild-type mice. (A) Reduced unc-79 mRNA expression in Lwt/Lwt mutant mice. 5.0 µg poly A (+) RNA from wild-type adult and postnatal day 0 (P0) Lwt/Lwt and wild-type whole brain was probed for unc-79 (upper portion) and β-actin (lower portion). Unc-79 expression is lower in Lwt/Lwt mutants, presumably due to nonsense-mediated decay. (B) Expression of unc-79 mRNA is restricted to nervous tissue in adult mice. An RNA blot containing poly A (+) RNA from various adult mouse tissues was hybridized sequentially with probes for unc-79 (upper portion) and β-actin (lower portion). The position of the markers is shown on the left side of the panels. See Text S1 for Materials and Methods. (0.48 MB TIF) [file pgen.1001057.s001.tif]

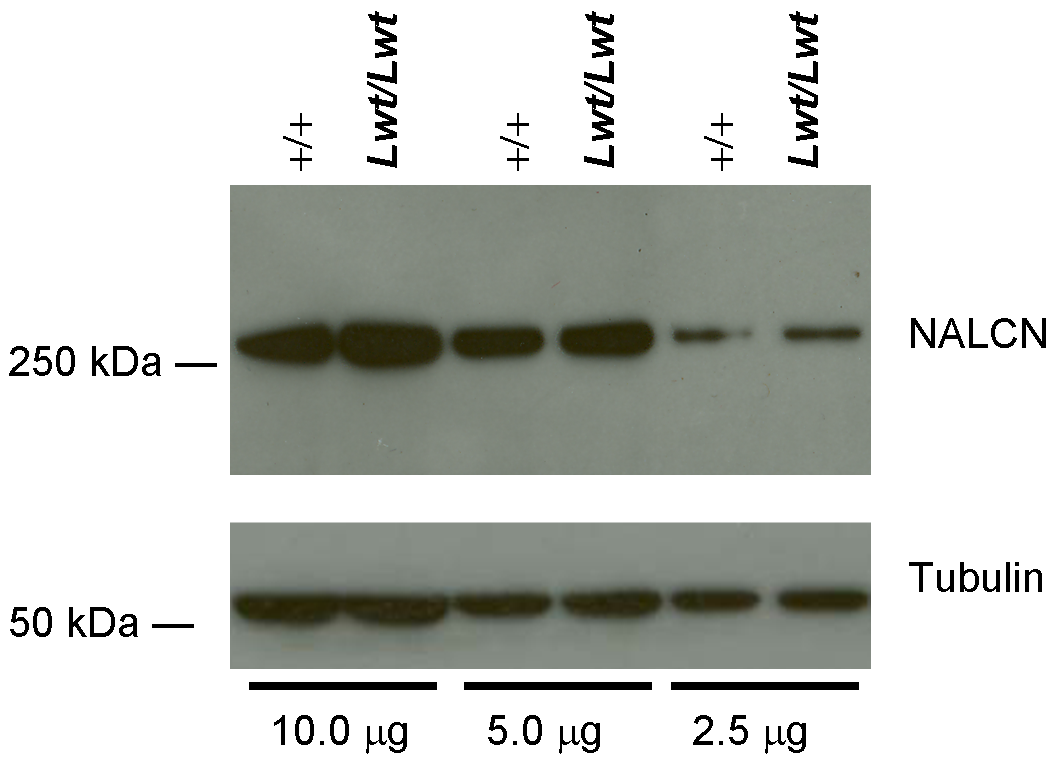

Supplement: Figure S2 — Western blot analysis of NALCN expression in Lwt/Lwt mice. Serial dilutions of postnatal day 0 (P0) brain lysates from a Lwt/Lwt pup and a +/+ littermate were blotted with rabbit α-NALCN polyclonal antibodies. There was no obvious alteration in expression of the NALCN protein. α-Tubulin antibodies were used as a loading control. (0.29 MB TIF) [file pgen.1001057.s002.tif]

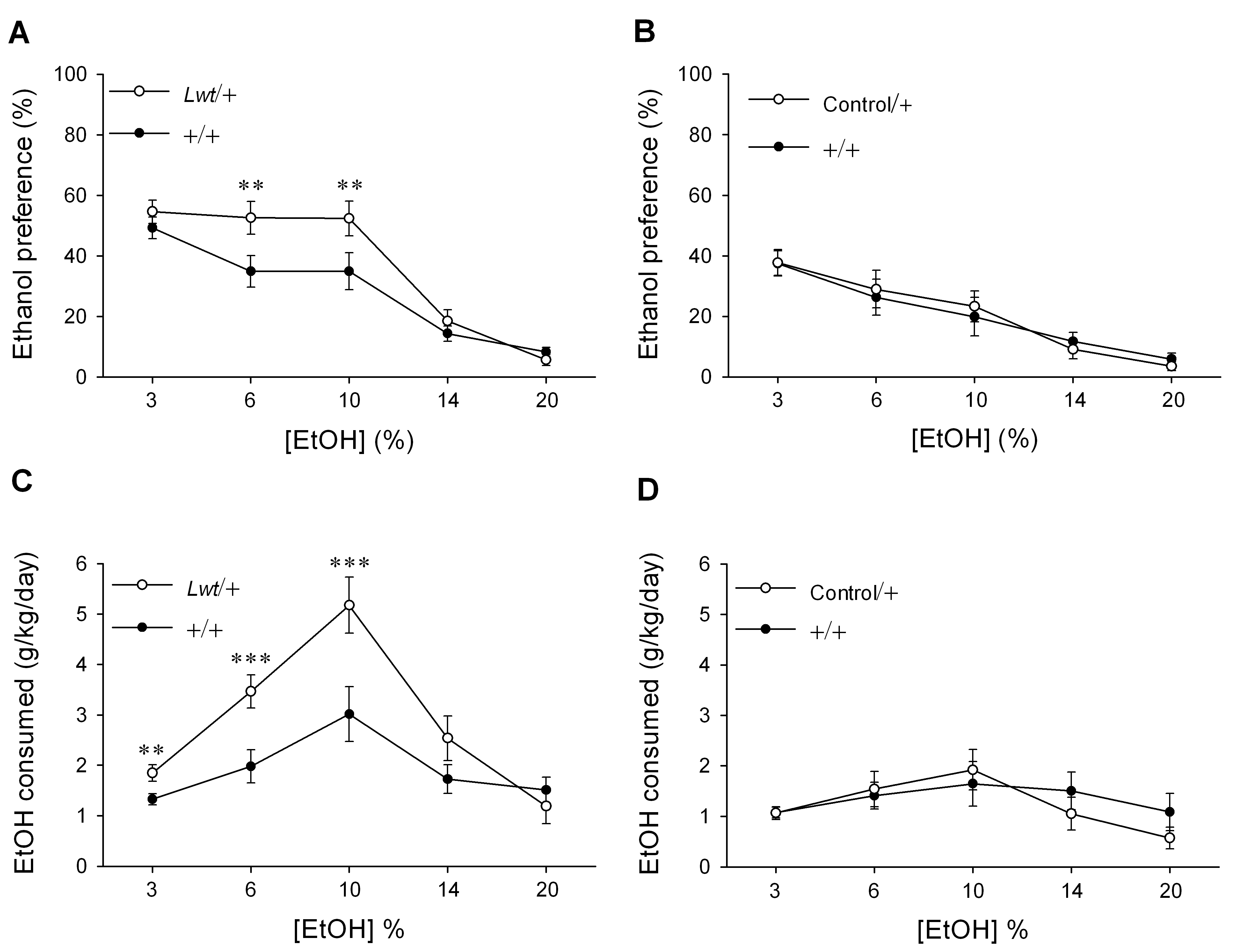

Supplement: Figure S3 — On a B6D2 F1 background, Lwt/+ congenic mice exhibit a higher preference for and consumption of ethanol in a two bottle choice test. (A) Lwt/+ (n = 19) animals have a higher preference for ethanol than +/+ littermates (n = 20) [F1,148(genotype) = 3.6, P = 0.064; F4,148(genotype × concentration) = 3.7, P = 0.007]. (B) There was no difference in ethanol preference in Control/+ (n = 16) relative to +/+ (n = 15) littermates [F1,116(genotype) = 0.01, P = 0.93; F4,116(genotype × concentration) = 0.26, P = 0.90]. (C) Lwt/+ (n = 19) animals consume more ethanol than +/+ littermates (n = 20) [F1,148(genotype) = 9.8, P = 0.003; F4,148(genotype × concentration) = 9.8, P<0.001]. (D) There was no difference in ethanol consumption in Control/+ (n = 16) relative to +/+ (n = 15) littermates [F1,116(genotype) = 0.17, P = 0.69; F4,116(genotype × concentration) = 0.97, P = 0.43]. (**P<0.01; ***P<0.001, post hoc Tukey test). (0.30 MB TIF) [file pgen.1001057.s003.tif]

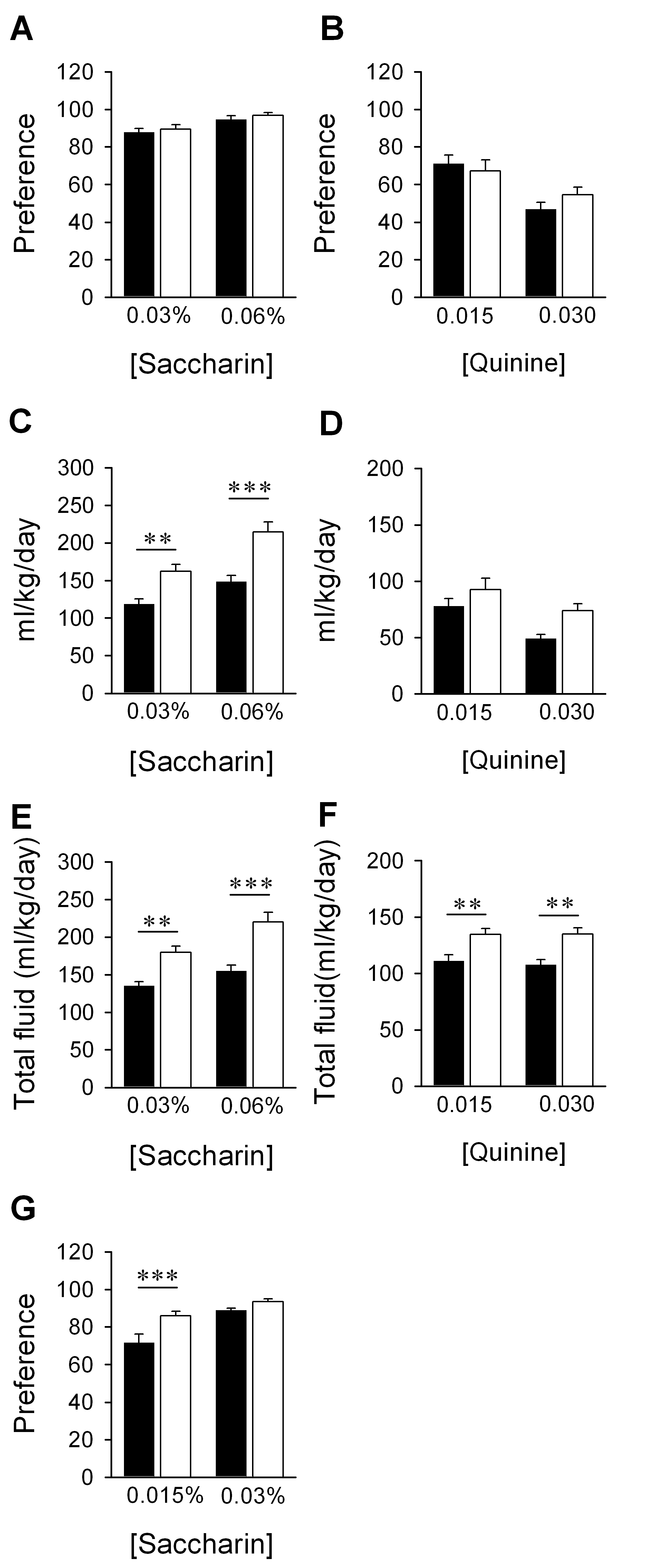

Supplement: Figure S4 — On a B6D2 F1 background, Lwt/+ congenic mice exhibit alterations in taste sensitivity and fluid consumption. (A) Relative to +/+ littermates (n = 20) (black bars), Lwt/+ (n = 18) (white bars) animals do not have altered preference for saccharin 0.03% or 0.06% [F1,36(genotype) = 0.70, P = 0.41] or (B) quinine (0.015mM or 0.030mM) [F1,36(genotype) = 0.09, P = 0.75]. (C) Consumption of saccharin is increased in Lwt/+ mice [F1,36(genotype) = 15.8, P<0.001; F1,36(genotype×concentration) = 8.2, P = 0.007]. (D) Consumption of quinine is not increased in Lwt/+ mice [F1,36(genotype) = 3.7, P = 0.061; F1,36(genotype × concentration) = 2.1, P = 0.155]. (E) Increased total fluid consumption contributes to the increased saccharin consumption [F1,36(genotype) = 20.6, P<0.001; F1,36(genotype × concentration) = 7.4, P = 0.010]. (F) Increased total fluid consumption is also observed for quinine-containing solutions [F1,36(genotype) = 11.9, P<0.001]. (G) A separate set of Lwt/+ animals (n = 12) and +/+ littermates (n = 12) were tested with a lower dose of saccharin (0.015%) and we observed a significant difference (***P<0.001, post hoc Tukey test) in preference at this concentration, suggesting that both the taste of saccharin and overall fluid consumption are altered in Lwt/+ mice on this background. (**P<0.01; ***P<0.001, post hoc Tukey test). (0.35 MB TIF) [file pgen.1001057.s004.tif]
