# Supplementary material for: Conserved Role of unc-79 in Ethanol Responses in Lightweight Mutant Mice
Source: PLoS Genet. 2010 Aug 12;6(8):e1001057. doi: 10.1371/journal.pgen.1001057 (PMC2920847; doi:10.1371/journal.pgen.1001057)
Supplement: Text S1 — Supporting Materials and Methods. (0.02 MB DOC) [file pgen.1001057.s005.doc]

**Supporting Materials and Methods**

***RNA analyses.*** Adult mouse poly A (+) mRNA was obtained from Ambion (Austin, Tx). Total RNA was isolated from wild type and *Lwt/Lwt* P0 whole brain tissue using Trizol reagent (Invitrogen), and poly A (+) mRNA was subsequently purified using a Fast Track poly A isolation kit (Invitrogen). 5.0 μg of poly A (+) mRNA was separated on a formamide gel, using reagents and protocols from Ambion (NorthernMax). A premade multiple tissue Northern blot (BD Biosciences/Clontech, #7762-1) containing ~ 2 μg poly A (+) RNA/tissue was probed for expression of the unc-79 transcript. A 1115 basepair probe of the *unc-79* transcript (including exons 17 thru 25) was used for detection. Blots were stripped and reprobed using an actin control probe (Ambion).

***Western analysis of NALCN protein.*** Affinity-purified rabbit α−mouse NALCN polyclonal antibodies were generously provided by Dr. Howard Nash (NIH). Whole brain lysates from wild-type and *Lwt/Lwt* mutant P0 animals were electrophoresed on 3-12% reducing Tris-Acetate gels and transferred to a nitrocellulose membrane (Invitrogen, Carlsbad, CA). After incubation with primary antibody, a goat anti-rabbit HRP-conjugated secondary antibody and ECL reagent (GE Healthcare) was used for detection. α−tubulin antibody was used as a loading control.
